# Abscisic acid induced freezing tolerance in chilling-sensitive suspension cultures and seedlings of rice

**DOI:** 10.1186/1756-0500-6-351

**Published:** 2013-09-03

**Authors:** Reiko Shinkawa, Aiko Morishita, Kumiko Amikura, Rika Machida, Hiroki Murakawa, Kazuyuki Kuchitsu, Masaya Ishikawa

**Affiliations:** 1 Division of Plant Sciences, National Institute of Agrobiological Sciences, Kannondai 2-1-2, Tsukuba 305-8602, Ibaraki, Japan; 2 Applied Biological Science, Graduate School of Science and Technology, Tokyo University of Science, Yamazaki 2641, Noda 278-8510, Chiba, Japan

**Keywords:** ABA (abscisic acid), Cold hardiness, Cell culture, Freezing injury, Freezing tolerance, Chilling injury, Rice (*Oryza sativa*)

## Abstract

**Background:**

The role of abscisic acid (ABA) as a possible activator of cold acclimation process was postulated since endogenous levels of ABA increase temporarily or constitutively during cold-hardening. Exogenous application of ABA has been known to induce freezing tolerance at ambient temperatures in *in vitro* systems derived from cold hardy plants. Yet, some cell cultures acquired much greater freezing tolerance by ABA than by cold whilst maintaining active growth. This raises questions about the relationships among ABA, cold acclimation and growth cessation. To address this question, we attempted to 1) determine whether exogenous ABA can confer freezing tolerance in chilling-sensitive rice suspension cells and seedlings, which obviously lack the mechanisms to acquire freezing tolerance in response to cold; 2) characterize this phenomenon by optimizing the conditions and compare with the case of cold hardy bromegrass cells.

**Results:**

Non-embryogenic suspension cells of rice suffered serious chilling injury when exposed to 4°C. When incubated with ABA at the optimal conditions (0.5-1 g cell inoculum, 75 μM ABA, 25-30°C, 7–10 days), they survived slow freezing (2°C/h) to −9.0 ~ −9.3°C (LT_50_: 50% killing temperature) while control cells were mostly injured at −3°C (LT_50_: -0.5 ~ −1.5°C). Ice-inoculation of the cell suspension at −3°C and survival determination by regrowth confirmed that ABA-treated rice cells survived extracellular freezing at −9°C. ABA-induced freezing tolerance did not require any exposure to cold and was best achieved at 25-30°C where the rice cells maintained high growth even in the presence of ABA. ABA treatment also increased tolerance to heat (43°C) as determined by regrowth. ABA-treated cells tended to have more augmented cytoplasm and/or reduced vacuole sizes compared to control cultures with a concomitant increase in osmolarity and a decrease in water content. ABA-treated (2–7 days) *in vitro* grown seedlings and their leaves survived slow freezing to −3°C with only marginal injury (LT_50_: -4°C) whereas untreated seedlings were killed at −3°C (LT_50_: -2°C).

**Conclusions:**

The results indicate that exogenous ABA can induce some levels of freezing tolerance in chilling-sensitive rice cells and seedlings, probably by eliciting mechanisms different from low temperature-induced cold acclimation.

## Background

Temperate cold hardy plants cold-acclimate or acquire cold hardiness (resistance to subfreezing temperatures; for more precise definitions, please see the Definition Section) in response to low temperatures alone or in combination with short photoperiods [1]. The molecular mechanisms involved in the induction of cold hardiness at the cellular level are still not well understood. Several studies have suggested that ABA may be involved in the initiation of cold acclimation. Endogenous levels of ABA have been demonstrated to increase temporarily during the initial stages of cold hardening [2-4] or constitutively during cold acclimation of cold hardy species [5]. Exogenous application of ABA is known to increase freezing tolerance of cold hardy plants, suspension cultures and callus cultures derived from plants capable of cold acclimation [6-9]. These findings have led to the hypothesis that cold acclimation is activated through the action of ABA; i.e. that low temperature brings about an increase in ABA, which triggers the activation of cold hardiness mechanisms [6].

In accordance with this hypothesis, ABA-deficient mutant (*aba1*) and ABA-insensitive mutant (*abi1*) of *Arabidopsis* plants following exposure to cold-acclimating conditions were less cold hardy compared to wild type plants [10-12]. But the results have to be interpreted with caution as these mutants have much less vigor than wild-type plants, which may result in lower capability of cold acclimation [13]. Analyses of COR gene expression in these mutants and wild-type plants revealed that some COR genes were highly responsive to exogenous ABA but their expression by low temperature was not necessarily mediated by ABA [11,14]. More recently, molecular analyses of low temperature-responsive genes in *Arabidopsis* have revealed that there are ABA-dependent and ABA-independent transcriptional pathways [15,16] and even cross-talks between these pathways [17]. The role of ABA in activation of low temperature responses is considered to be minor than it was thought [13]. Yet, questions still remain unanswered as to how ABA alone can induce high levels of freezing tolerance in some plant systems and how it should be interpreted, especially with regard to cold-induced freezing tolerance.

Induction of freezing tolerance by exogenous ABA in cold hardy bromegrass suspension cells has attracted attention as it can induce high levels of freezing tolerance (LT_50_: -28 ~ −35°C) at non-hardening temperature (25-30°C) in a rather short period of time [7,18]. Not only freezing tolerance, heat, salt and osmotic stress tolerance were simultaneously induced by ABA (cross-adaptation) [18]. Comparison of low temperature-induced freezing tolerance and ABA-induced freezing tolerance may provide a unique approach to understanding cold hardiness mechanisms. Physiological, morphological analyses [7], gene expression and protein analyses [19-21] have all shown that ABA-induced freezing tolerance is different from the one induced by low temperature. Ishikawa et al. [7] considered that behavior of bromegrass cells during induction of freezing tolerance by ABA was similar to that of the seed formation process.

Rice originates from tropical and subtropical areas and is sensitive to chilling temperatures (cool temperature ranges above 0°C) at various developmental stages such as booting, flowering and seedling stages [22,23]. Seedlings suffer injuries upon exposure to 5-10°C for 3–11 days [24-26]. Callus cultures of rice are also known to suffer chilling injury at 5°C [27]. Since rice plants suffer injuries at cool temperature ranges suitable for cold-hardening of cold-hardy plants, they are considered unable to acclimate to cold and to be freezing-sensitive [25]. More recently, it has been demonstrated that rice is capable of reducing chilling injuries (4–7 days exposure to 4°C) by prior exposure to 12°C for 2 days, more pronouncedly in japonica cultivars than indica cultivars [28]. However, this does not mean that rice can withstand prolonged exposure to chilling temperatures or can further acclimate to freezing temperatures. This can be easily proven by observing japonica rice cultivars in field conditions at Tsukuba, ca. 50 km north-east of Tokyo, Japan. After harvesting in the early autumn, new sprouts come out of the remaining rice stubble and grow to 10–15 cm tall in warm temperatures of October and November. These shoots seem to withstand transient exposure (daily or for several days) to chilling temperatures during this period but they are likely unable to acquire freezing tolerance in the autumnal conditions. They are eventually killed either by prolonged exposure to chilling temperatures and/or by frosts in late November or early December and are unable to overwinter in this area (none of them can regrow in the following spring).

It is of interest to check whether chilling-sensitive plants that lack the capability of cold acclimation (acquire freezing tolerance in response to cold) can attain freezing tolerance in response to ABA. To our knowledge, there has been no such attempt. This may enhance our understanding of ABA-induced freezing tolerance.

The objective of this study is to determine whether ABA can induce freezing tolerance in rice, a chilling-sensitive plant, which lacks low temperature-induced cold acclimation capability. The study was also intended to optimize culture conditions for inducing maximal freezing tolerance by ABA in rice and to physiologically characterize this system. The results were compared with cold-hardy bromegrass suspension cells to help understand the nature of ABA-induced freezing tolerance.

## Results

### Chilling sensitivity of rice suspension cells and effect of ABA

**Figure 1:**
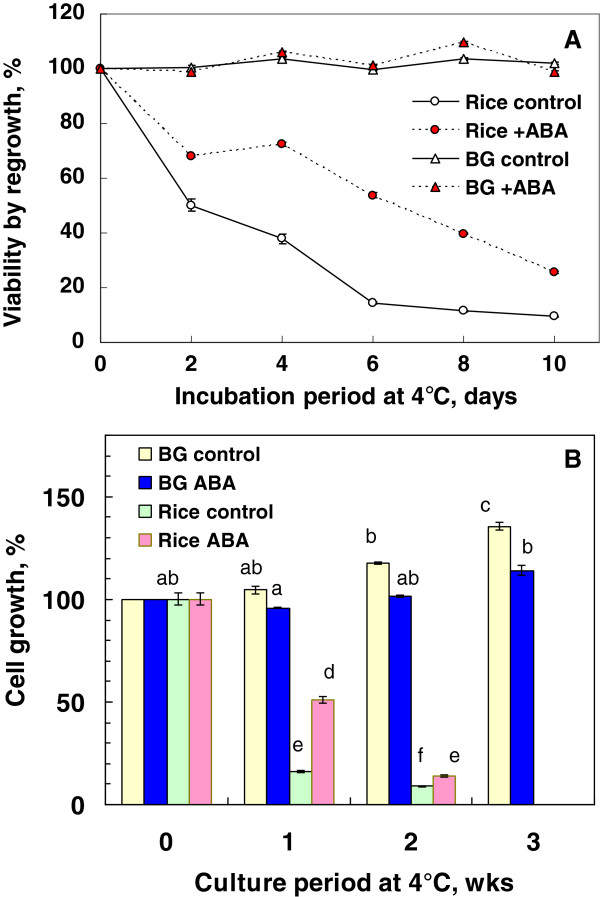
**Chilling sensitivity of suspension cells of rice and bromegrass cultured at 4°C in the presence or absence of 75 μM ABA (A) and growth of rice and bromegrass cells during prolonged culture at 4°C with or without 75 μM ABA (B).** Rice or bromegrass cells (0.25 g) inoculated in 12.5 mL of N6 medium or ER medium were incubated at 4°C in the presence or absence of 75 μM ABA for designated periods following 2 day pre-incubation at 25°C. Survival of chilled cells was determined by regrowing the cells (non-stressed cells as the 100% control). Values indicated by different letters (a-f) were significantly different (α = 0.05) using Tukey-Kramer multiple comparison analyses (Figure 1B).

To check chilling sensitivity of rice suspension cells, cells were exposed to 4°C for up to 10 days in the presence or absence of 75 μM ABA. Bromegrass suspension cells were also incubated in the same manner as a chilling tolerant control. Exposure of rice suspension cultures (without ABA treatment) to 4°C for more than 2 days greatly reduced the viability as determined by regrowth capacity, which indicated a high level of chilling-sensitivity of rice cells (Figure 1A). Inclusion of ABA (75 μM) in the medium reduced the chilling injuries of rice cells but only 30% of ABA-incubated cells survived after 10 days of incubation at 4°C. In contrast, suspension cells of cold-hardy and chilling tolerant bromegrass did not suffer any injuries during 10 day-incubation at 4°C both in the presence and absence of ABA (Figure 1A). Continuous incubation of rice suspension cultures at 4°C resulted in drastically diminished growth in the 3 week period whilst that of bromegrass cultures increased fresh weight by 14% (+ABA) and 36% (−ABA) after 3 weeks (Figure 1B).

### Effect of ABA on freezing tolerance of rice suspension cells

**Figure 2:**
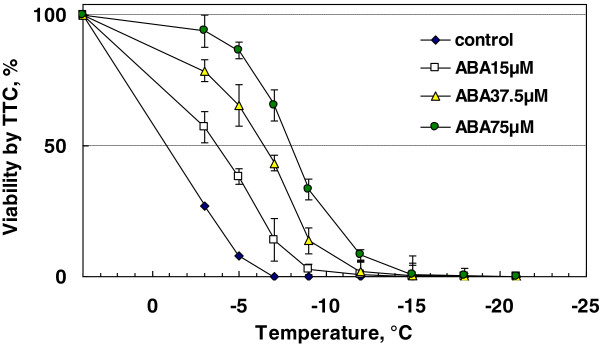
**Freeze survival of rice suspension cells cultured in the presence of ABA (0–75 μM) for 7 days at 25°C (1 g cell inoculum in 50 mL of medium).** Freezing tests were done as described in the Methods and survival was determined by TTC (2,3,5-triphenyl tetrazolium chloride) reduction assays.

To determine whether rice suspension cells can acquire freezing tolerance (not chilling tolerance) by incubation with ABA, rice cultures (initiated from 1 g of cell inoculum) were incubated at 25°C for 7 days in the presence of 0, 15, 37.5, 75 μM ABA and harvested cells were used for freeze tests. Increasing concentrations of ABA allowed the cells to acquire higher levels of survival at freezing temperatures (Figure 2). The highest freezing tolerance (LT_50_: -8.0°C) as determined by TTC reduction tests was obtained with 75 μM ABA. Incubation of rice cells with 100 μM ABA conferred a similar level of freezing tolerance (LT_50_: -7.9°C) and 75 μM ABA was likely the optimal concentration for inducing freezing tolerance in rice cells. This is similar to the optimal concentrations of other plant systems where ABA-induced cold hardiness was attempted [6-9]. Regrowth assays gave essentially similar freeze survival results (data not shown). At the beginning of the freeze tests (−3°C), each test tube was ice-inoculated and equilibrated for 1 h before further cooling. Freezing of the cells in the medium (water) inside the tube was visually ascertained at this step and also at the designated temperature when the tube was taken out of the freezer. Thus the cell survival observed at freezing temperatures was not due to incidental supercooling but to tolerance of extracellular freezing at a slow cooling rate (2°C/h).

### Time course analysis of freezing tolerance induction by ABA

**Figure 3:**
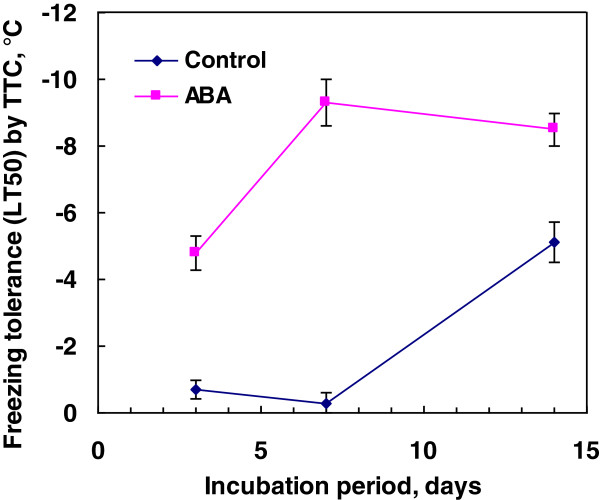
**Time course dependent changes in freezing tolerance (LT**_**50**_**) of rice suspension cells incubated with or without 75 μM ABA at 25°C (1 g cell inoculum in 50 mL of medium).** Freezing tolerance (LT_50_) was determined by plotting the survival at each subfreezing temperature determined by TTC reduction assay.

Chronological changes in freezing tolerance were followed with rice suspension cells cultured with or without 75 μM ABA for up to 14 days. Incubation with ABA induced freezing tolerance (LT_50_) of −4.8°C in 3 days as determined by TTC assay (Figure 3). Induced level of freezing tolerance increased to −9.3°C in 7 days and was maintained until 14 days (−8.5°C). In contrast, the control cells harvested on day 3 and 7 did not tolerate freezing (LT_50_: -0.7 and −0.3°C, respectively) whilst on day 14 the cells acquired freezing tolerance of −5.1°C. Similar acquisition of freezing tolerance in control cells at the stationary stage of the growth curve has been observed in bromegrass suspension cells [7,29].

### Effect of cell inoculum size on ABA-induced freezing tolerance

**Figure 4:**
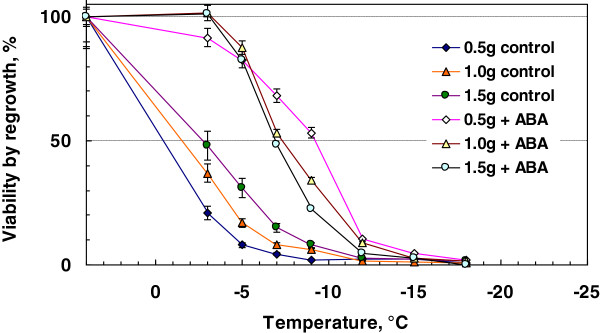
**Effect of cell inoculum size on freezing tolerance of rice cells incubated with or without ABA.** Cell cultures initiated from 0.5-1.5 g of rice cells in 50 mL of medium were incubated at 25°C for 7 days with or without ABA (75 μM). Freezing tolerance was determined as described in the Methods and survival was determined by regrowth.

In the case of freezing tolerance induction by ABA in bromegrass suspension cultures, the amount of cells inoculated in the medium was one of the most influential factors determining the level of induced freezing tolerance [7]. To see whether this holds true with rice suspension cells, cultures initiated from 0.5, 1 and 1.5 g cells were grown in the presence or absence of 75 μM ABA for 7 days at 25°C and the freezing tolerance induced was compared. The highest freezing tolerance (LT_50_: -9.0°C) as determined by regrowth capacity was obtained when culture was initiated from 0.5 g cells, followed by 1 g and 1.5 g (Figure 4). Control cultures of rice suspension cells grown without ABA showed the lowest freeze survival (LT_50_: higher than −3°C) when culture was initiated from 0.5 g cells, followed by 1 g and 1.5 g (Figure 4). The greatest difference in freeze survival between ABA-treated cultures and control cultures was obtained with cultures initiated from 0.5 g of cells.

### Effect of incubation temperature on ABA-induced freezing tolerance

**Figure 5:**
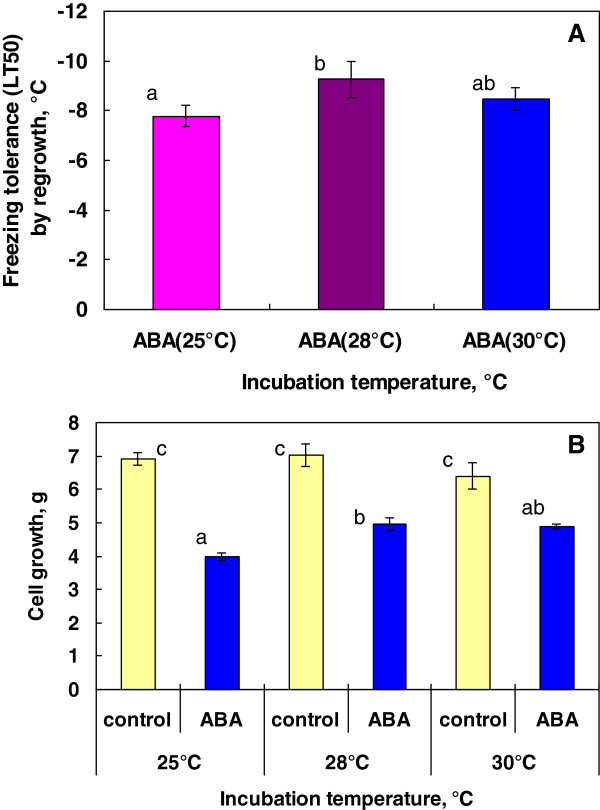
**Effect of culture temperatures on freezing tolerance induced by exogenous ABA (A) and growth (B) of rice suspension cells.** Cell cultures (1 g cell inoculum in 50 mL medium) were incubated at 25, 28 or 30°C for 7 days with or without 75 μM ABA. Freeze survival was determined by regrowth and cell growth on fresh weight basis was measured as detailed in the Methods. Values indicated by different letters (a-c) significantly differed at 5% level using Tukey-Kramer multiple comparison analyses.

In the case of bromegrass suspension cultures, the optimal temperature for inducing freezing tolerance by ABA was found to occur at 25 and 30°C among the temperatures tested (5, 10, 15, 20, 25, 30°C) and the level of freezing tolerance induced was proportionally related to the amount of growth achieved [7]. To check whether this holds true with rice suspension cells, rice suspension cells (initiated from 1 g of cells) were grown for 7 days with 75 μM ABA at different incubation temperatures (25, 28, 30°C). The highest freezing tolerance (LT_50_: -9.3°C) was obtained at 28°C, followed by 30°C (LT_50_: -8.5°C) then 25°C (LT_50_: -7.9°C) (Figure 5A). At these temperatures, rice cells showed considerable growth in the presence of ABA (but not as much as the control cells) and the level of ABA-induced freezing tolerance was greater where there was more growth (28 > 30°C > 25°C) (Figure 5B).

### Effect of light, shaker speed and flask size on ABA-induced freezing tolerance

**Figure 6:**
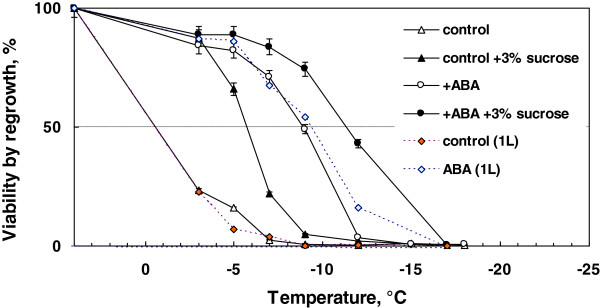
**Effect of 3% (w/v) of sucrose included in the freezing medium on freezing tolerance of rice suspension cells.** Cell cultures (0.5 g cell inoculum in 50 mL medium) were incubated at 28°C for 7 days with or without 75 μM ABA. Harvested cells were frozen either in 0.1 mL of 3% sucrose or in 0.1 mL of sterile water. Freeze survival was determined by regrowth. Freeze survival of cell cultures (2.5 g cell inoculum in 1 L medium) incubated at 28°C with or without 75 μM ABA (no sucrose in the freezing medium) was also shown for comparison.

Oxygen supply is an important factor for cold acclimation as it requires ATP and NAD(P)H [1]. Shaker speed may limit oxygen supply to suspension cells but culture agitation between 85 and 120 rpm (1 g cell inoculum in 50 mL of medium at 25°C) did not greatly influence the level of freezing tolerance induced by ABA (data not shown). To check whether there is any effect of flask sizes that may also affect oxygen supply to suspension cells, 2.5 g of cells were inoculated into 250 mL of medium with or without ABA in 1 L flasks, instead of 0.5 g of cells inoculated into 50 mL of medium in 200 mL flasks (the same cell inoculum/medium ratio). However, the use of 1 L flask altered the level of ABA-induced freezing tolerance (LT_50_) only marginally (−9.3°C) compared to the 200 mL flask system (LT_50_: -9.0°C) as determined by regrowth assays (Figure 6). An improved survival (16%) at −12°C was obtained by the use of 1 L flask system.

### Maximal freeze survival induced by ABA and effect of inclusion of 3% sucrose in the freezing medium on freezing tolerance

The results presented in Figures 4 and 5 imply that a better freeze survival may be obtained by incubation of 0.5 g cells at 28°C in 50 mL medium (+ 75 μM ABA). However, there was no further improvement in the freezing tolerance (LT_50_: -9.0°C) under this condition (Figure 6). These results suggest that the maximal freezing tolerance level acquired by ABA treatment in rice suspension cells are in the range of −9 ~ −9.3°C.

Exogenous sucrose is known to be a good cryoprotectant [1]. We investigated whether this is also the case with rice suspension cells. The use of 3% sucrose, instead of water, for the freezing medium improved the level of freezing tolerance of control (LT_50_: -5.7°C) and ABA-treated rice cells (LT_50_: -11.3°C) compared to freezing tolerance determined in plain water (LT_50_ of control and ABA-treated cells: -0.6 and −9.0°C, respectively) as assayed by regrowth.

### Effect of ABA on heat tolerance of rice suspension cells

**Figure 7:**
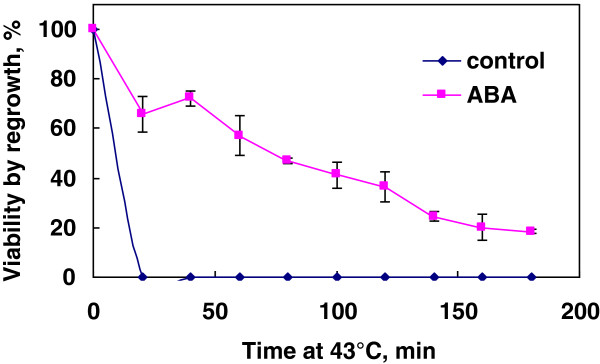
**Heat tolerance of rice suspension cells incubated in the presence or absence of 75 μM ABA for 7 days at 25°C (1 g cell inoculum).** Survival of cells directly exposed to 43°C for 0–180 min (without heat shock pretreatment) was determined by regrowth capacity compared to that of non-stressed cells.

To check whether ABA can induce stress tolerance other than freezing, heat tolerance was determined with rice cells grown for 7 days with or without 75 μM ABA. The harvested cells were exposed directly to 43°C for 0 to 180 min without heat shock pretreatment. ABA-treated cells showed much higher survival throughout the treatment period (20–180 min) compared to control cells as determined by regrowth capacity (Figure 7).

### Changes in physiological and morphological parameters of rice cells induced by ABA

**Figure 8:**
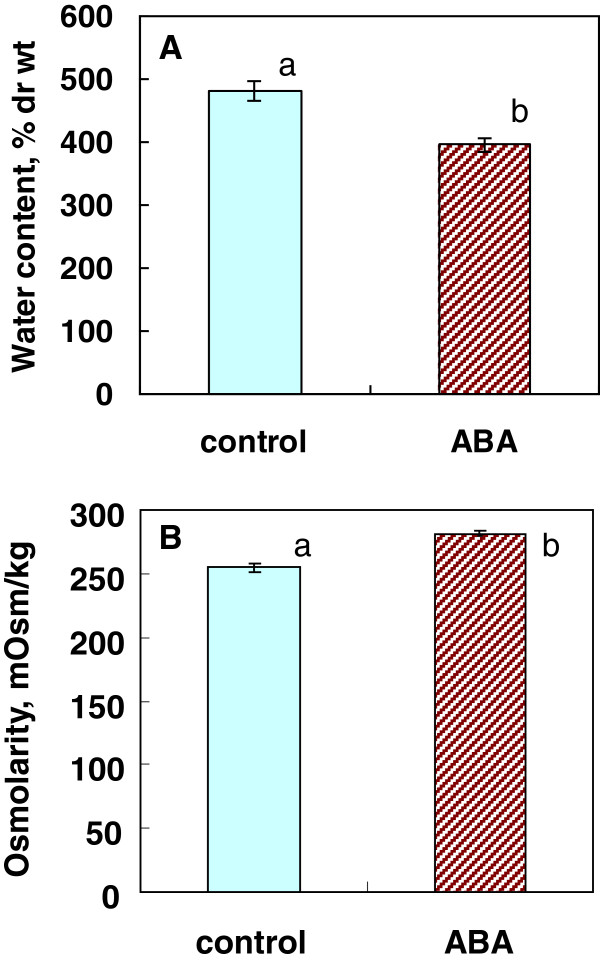
**Effect of ABA on water content (A) and osmolarity (B) of rice suspension cells.** Rice suspension cultures were incubated at 25°C for 7 days in the presence or absence of 75 μM ABA before being processed for determining these parameters as detailed in the Methods. Values indicated by different letters (a-b) were significantly different (α = 0.05) using Tukey-Kramer multiple comparison analyses.

To reveal physiological changes induced by ABA treatment, we determined osmolarity and water content of rice cells incubated with or without ABA for 7 days at 25°C. Since suspension cells were incubated in liquid media, the cells were rich in the intercellular water, which was not easy to remove completely. We centrifuged the harvested rice cells at 1500 rpm for 10 min to remove the majority of the intercellular water prior to determination of water content and osmolarity. ABA-treated cells had a water content of 395% on dry weight basis, which was ca. 18% reduction compared to the control cells (481% dry weight) (Figure 8A). Meanwhile, the ABA-treated cells had a slightly (by 11%) increased osmolarity (282 mOsm/kg) compared to the control cells (255 mOsm/kg) (Figure 8B). Osmolarity in this range likely contributes to freeze point depression of only 0.5°C, which is not sufficient for avoiding freezing of cellular water at −3°C or lower. The difference in the osmolarity was not large enough to explain the differences in freezing tolerance between control and ABA-treated cells.

**Figure 9:**
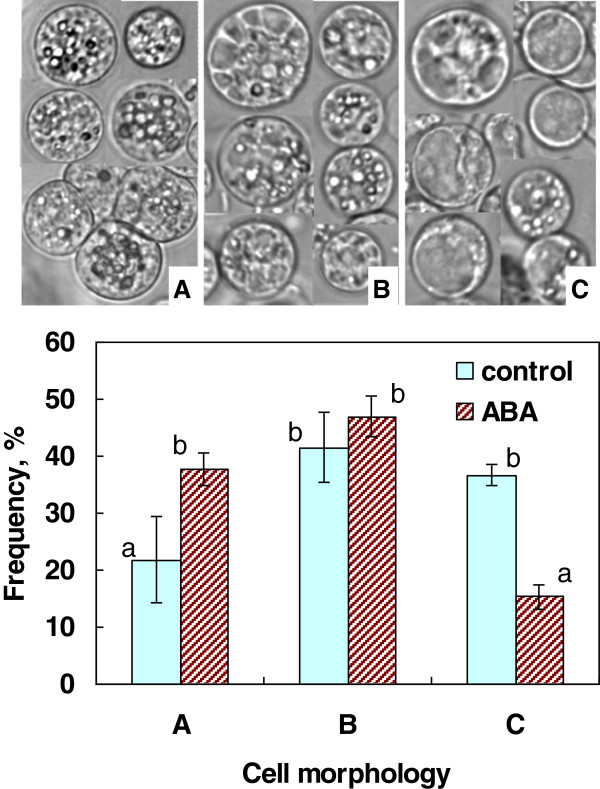
**Differences in cellular morphology types (A-C) of protoplasts isolated from rice suspension cells grown in the presence or absence of ABA at 25°C for 7 days.** Typical examples of protoplasts for each morphology category (**A**: augmented; **B**: less vauolated; **C**: highly vacuolated) were shown on top of the graph. Values indicated by different letters (a-b) were significantly different (α = 0.05) using Tukey-Kramer multiple comparison analyses.

To determine any changes in the cellular structure induced by ABA treatment, we isolated protoplasts from the rice cells incubated with or without 75 μM ABA for 7 days at 25°C for observation under microscopy. ABA-treated cultures were rich with cells that had more augmented cytoplasm with increased particles and/or reduced vacuole sizes whereas control cultures had a higher frequency of well-vacuolated cells (Figure 9).

### Effect of ABA on freezing tolerance of *in vitro* grown rice seedlings

**Figure 10:**
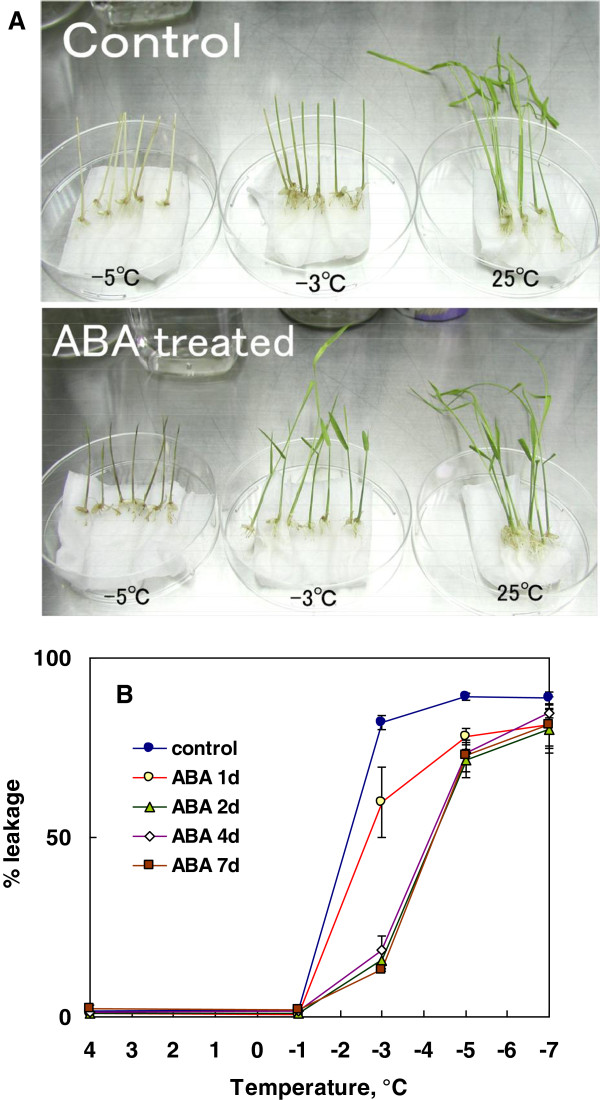
**Effect of ABA on freezing tolerance of *in vitro *grown rice seedlings.** Rice seedlings were grown aseptically *in vitro* for 8 days under normal conditions at 25°C followed by 1–7 day-incubation in the presence of 75 μM ABA and used for freeze tests as described in the Methods. Survival was determined by regrowth of the seedlings (**A**: the result of 4 day-incubation with ABA) and by electrolyte leakage of detached leaves **(B)**.

To see whether ABA can induce freezing tolerance in plant systems other than cell cultures, we determined freezing tolerance of young rice seedlings and their leaves treated with or without ABA. When leaves of control rice seedlings (grown for 8 days at 25°C) were exposed to slow freezing to −3°C following ice-inoculation at −1°C, 82% of the tissues were injured (LT_50_: -2°C) as shown by leakage tests (Figure 10B). Leaves of seedlings incubated with ABA for 2 days or more had reduced injuries (15%) when slowly frozen to −3°C but showed over 70% injury at −5°C (LT_50_: -4°C). In accordance with leakage test results, whole rice seedling that had been incubated with 75 μM ABA 4 days survived slow freezing to −3°C with the ability to regrow whilst they were unable to regrow after exposure to −5°C (Figure 10A). In contrast, control rice seedlings (without ABA treatment) exposed to slow freezing to −3°C were unable to regrow although they retained some green in the leaf sheath (Figure 10A). In the freeze tests, each test tube was ice-inoculated at −1°C and held there for 30 min or 1 h before further cooling. Freezing of the tissues in suspension medium (water) was visually ascertained at this step and also at the designated temperature when the tube was taken out of the freezer. Thus the tissue survival at freezing temperatures was not due to incidental supercooling or to freeze point depression, but to tolerance of slow extracellular freezing (cooling rate: 2°C/h). The results indicated that ABA can induce some levels of freezing tolerance in rice seedlings although the level of tolerance induced was less than the case of cell cultures.

## Discussion

The results clearly indicate that ABA can induce some level of freezing tolerance in suspension cultures and seedlings of rice. The levels of maximal freezing tolerance induced (LT_50_) were −9°C in cultured cells and −4°C in seedlings, which were about 7-8°C and 2°C below the LT_50_ of the respective control. The observed survival was not due to temporary supercooling or freezing point depression but to the tolerance of slow extracellular freezing and ascertained by regrowth capability as well as TTC reduction tests and electrolyte leakage tests. Induced freezing tolerance in suspension cells was more than that (LT_50_: -6.5 ~ −8°C) of *in vitro* grown *Arabidopsis* plants after 4–8 days of cold acclimation at 2-4°C [10,11]. Rice seedlings have been known to be chilling-sensitive and unable to undergo cold acclimation in response to temperatures slightly above 0°C [25]. Our results confirm that rice suspension cells are seriously injured by exposure to 4°C (Figure 1). To our knowledge, this is the first paper describing that ABA can confer freezing tolerance in such a chilling-sensitive plant system. Many studies have shown that prior exogenous application of ABA reduced the level of chilling injuries in chilling-sensitive plants (e.g., [30,31]), but none of them attempted to measure freezing tolerance of such chilling-sensitive plant systems treated with ABA.

In contrast to rice suspension cells, suspension cells derived from smooth bromegrass (cold hardy grass) were not only tolerant to exposure to 4°C but also able to resume growth at 4°C (Figure 1). At the optimal conditions, exogenous ABA induced extremely high levels of freezing tolerance (LT_50_: -28 ~ −35°C) in bromegrass cells [7,18]. Compared to bromegrass, the level of freezing tolerance induced (LT_50_: -9°C) was much less in rice suspension cells. Yet, detailed comparison of the conditions for ABA-induced freezing tolerance in rice and bromegrass systems allowed us to find many shared commonalities. For example, the level of ABA-induced freezing tolerance was influenced by incubation period (optimum: 7–10 days), ABA concentration (optimum: 75 μM or more) and cell inoculum size (optimal cell inoculum: 0.5-1 g / 50 mL medium) and was unaffected by light conditions, shaker speed (Figures 2, 3, 4, 5, 6). Induction of freezing tolerance by ABA in both systems occurred at non-hardening temperatures, having the optimum temperature at 25-30°C and did not require any exposure to low temperatures [7]. Both bromegrass and rice cells grew well in the presence of ABA at these temperatures (Figure 5). The highest level of ABA-induced freezing tolerance was achieved where the greatest growth was attained in both systems [7]. In bromegrass cells, ABA induced cross-adaptation conferring heat, osmotic, salt tolerance and freezing tolerance simultaneously [18]. In rice cells, it was shown that heat tolerance was increased by ABA besides freezing tolerance (Figure 7).

Microscopic observation of rice cells during this process revealed that ABA-treated cells tended to have more augmented cytoplasm with increased intracellular particles and/or reduced vacuole sizes compared to control cells (Figure 9). This was accompanied by concomitant increases of osmolarity and decreases of water content in ABA-treated cells (Figure 8) [7]. These characteristics of the cells are typical of cold-acclimated cells [1] but they are also typical of cells in developing and ripening seed embryos where ABA plays important roles [32]. As discussed above, both rice and bromegrass suspension cells acquire freezing tolerance at 25-30°C where they maintained high growth rates even in the presence of ABA. It is interesting to note that seed development and maturation proceed well in the presence of intrinsic ABA at 25-30°C [32,33]. Our recent proteomic analyses of rice suspension cells treated with or without ABA have revealed that ABA induced new sets of proteins in 2–7 days and protein profiles of ABA-treated cells were similar to that of rice seed embryos (manuscript in preparation).

As reviewed in the Background section, endogenous ABA has been known to increase during cold acclimation in cold hardy plants [2-5], which led to the hypothesis that ABA may work as an activator of triggering cold acclimation [6]. Molecular analyses of low temperature-responsive genes in *Arabidopsis* have revealed that there are at least ABA-dependent and ABA-independent transcriptional pathways [15,16] and even cross-talks between these pathways [17]. This may partly prove the contribution of ABA in cold acclimation and yet numerous questions still remained unanswered. In *Arabidopsis* suspension cells, for example, exogenous ABA treatment did not confer freezing tolerance (our unpublished data). In contrast, in some plant cell cultures derived from cold hardy plant species such as bromegrass and wheat, exogenous ABA induced much higher levels of freezing tolerance than did cold treatments [7,34]. Galiba et al. [34] found that exogenous ABA treatment allowed calli derived from various wheat cultivars to attain similarly high levels of freezing tolerance irrespective of freezing tolerance levels induced by cold treatment. Why do these things happen and how should they be interpreted? Does freezing tolerance induction by ABA and cold involve different mechanisms?

Our previous studies on ABA-induced freezing tolerance in bromegrass suspension cells have shown that ABA-induced freezing tolerance is different from the one induced by low temperatures. For instance, physiological conditions such as the optimal temperatures were different between ABA and cold-induced ones as discussed above and cell morphology was also different [7]. Great differences were observed in gene expression profiles [21] and protein profiles [19,20,35,36] between the two freezing tolerance induction systems. Ishikawa et al. [7] considered that a physiological process similar to seed formation/maturation proceeds in ABA-treated bromegrass cells, which confers various stress tolerance concurrently. Our current results clearly show that ABA can induce some levels of freezing tolerance in a chilling-sensitive plant system that lacks the ability to undergo cold acclimation. This will be another piece of evidence that ABA-induced freezing tolerance is elicited by mechanisms different from the low temperature-induced cold acclimation process, perhaps mimicking seed development and maturation. And yet, the level of freezing tolerance induced in rice by ABA was much less than the case of cold hardy bromegrass cells, the reasons of which require further investigation.

The level of freezing tolerance induced by ABA in rice seedlings was less (LT_50_: -4°C) than that in suspension cells (LT_50_: -9°C). This also holds true with cold hardy plant systems such as bromegrass or wheat cell cultures and their corresponding seedlings. Proteomic analyses revealed that similar proteins were induced in both rice cell cultures and seedlings treated with ABA (manuscript in preparation). Both in bromegrass [7] and rice suspension cells (Figure 5), cell cultures treated with ABA showed active growth at ambient temperatures and the level of freezing tolerance induced was proportional to the amount of growth. In contrast, ABA treatment retarded or stopped the growth of rice seedlings (data not shown). The limited levels of freezing tolerance acquired in ABA-treated seedlings may be related to the controlled or restricted manner of growth and differentiated functions of various types of cells in the seedlings. ABA-induced freezing tolerance may likely be best expressed in cultured cells or undifferentiated dividing cells if it involves a process similar to seed development and maturation.

## Conclusions

The results clearly indicate that exogenous ABA induces some levels of freezing tolerance (maximum LT_50_: -9°C and −4°C, respectively) in rice suspension cells and seedlings. The survival was not due to temporary supercooling but to the tolerance of slow extracellular freezing and ascertained by regrowth capability. ABA treatment also induced heat tolerance. The optimum conditions for ABA-induced freezing tolerance in rice cells and those in cold hardy bromegrass cells were similar. ABA-induced freezing tolerance was best achieved at 25-30°C where the cells maintained high growth even in the presence of ABA. This is probably the first paper reporting that ABA can confer some levels of freezing tolerance in a chilling sensitive plant system that lacks the ability to undergo cold acclimation. This provides further evidence that ABA-induced freezing tolerance is elicited by mechanisms different from the low temperature-induced cold acclimation process.

## Methods

### Cell cultures

Experiments were mainly conducted with a non-embryogenic cell suspension culture (line: OcN6) of rice (*Oryza sativa* L.), that had been derived from rice Oc suspension culture [37] and subcultured for more than two years following acclimation to N6 medium [38]. The original medium for the Oc culture was 2,4-D supplemented MS [37,39], but when we obtained this culture, the culture was maintained in a medium containing GA and BA, which are known to inhibit freezing tolerance induction by ABA [40]. Simpler media containing 2,4-D as the sole hormone were preferred. For this reason, the stock culture was transferred to N6 medium (OcN6) and maintained at 25°C on a rotary shaker (85 rpm) by biweekly subculturing in 50 mL of N6 medium (pH 5.8 with 3% w/v sucrose and 1 ppm 2,4-D).

In chilling experiments, a suspension culture of smooth bromegrass (*Bromus inermis* cv. Manchar) was used as the control representing cold hardy plant cells to compare with rice cells. Bromegrass suspension cells were maintained in ER medium (0.5 ppm 2,4-D) at 25°C as described previously [7].

### Culture conditions for ABA treatment

Individual experiments were initiated with one g fresh weight of rice suspension cells from 7 day-old stock cultures unless otherwise specified. In typical experiments, cells were incubated in 50 mL of medium with 75 μM ABA (treated) or without ABA (control) on a rotary shaker (85 rpm) at 25°C for 7 days.

To find optimal conditions for ABA-induced freezing tolerance, the effect of cell inoculum size (0.5, 1, 1.5 g), ABA concentration (0–75 μM), shaker speed (85, 100 and 120 rpm), incubation temperature (25, 28, 30°C), the presence of light (50 μmol s^-1^ m^-2^) and inclusion of 3% (w/v) sucrose in the freezing medium were checked. Cells were usually incubated with or without ABA for 7 days before determination of freezing tolerance. When necessary, time course-dependent changes in freezing tolerance were also determined.

### Chilling tests for suspension cells

Rice or bromegrass suspension cells (0.25 g inoculated in 12.5 mL of respective culture medium with or without 75 μM ABA) were pre-incubated at 25°C for 2 days (shaker speed: 85 rpm). Then the cells were incubated at 4°C for 0–10 days (shaker speed: 85 rpm) in the dark. Following the chilling treatment, the cells were re-incubated at 25°C for 5 days prior to harvest. The harvested cells were washed with ample water and cell dry weight was determined after oven-drying at 70°C for 2 days. Survival% was determined by regrowth of chilled cells compared to that of non-stressed cells and 100% dead cells as detailed elsewhere [18].

To unravel growth characteristics of bromegrass suspension cells during prolonged incubation at low temperature, bromegrass cultures (0.25 g cells in 12.5 mL of ER medium) were incubated at 4°C for 1–3 weeks in the presence or absence of ABA before determining fresh weight increases as compared to the initial cell inoculum.

### Freeze tests for suspension cells

Following incubation under designated culture conditions with or without ABA, cells from a flask were harvested by filtration and washed with 250 mL of sterilized distilled water to remove residual medium, which otherwise would affect freezing and heat tolerance levels [7,18]. For regrowth analyses, 0.25 g fresh weight of cells were placed in a 15 mL pre-sterilized tube with 0.1 mL of sterile water for each test temperature (triplicates). The cells were ice-nucleated at −3°C by touching the tube with dry ice. Frozen cells were held at −3°C for 1 h and then cooled at 2°C/h to −12°C, and from −12 to −21°C at 5°C/h. Cells were removed at designated test temperatures and thawed at 4°C prior to evaluating viability. Both control and ABA-treated cells were killed by direct submersion in liquid nitrogen followed by rapid thawing for several cycles. All these procedures were done aseptically.

In some freeze tests, rice cells were frozen in the presence of 3% (w/v) sucrose (0.1 mL) instead of sterile water and processed in the same manner to see the effect of sucrose on the freeze-survival.

### Heat tests for suspension cells

Cultures, initiated with 1 g fresh weight inocula in 50 mL N6 medium, were incubated at 25°C for 7 days with or without 75 μM ABA. Cell sampling was done as described in the freezing tolerance section except a heat stress was imposed by incubating cells in a water bath at 43°C from 0 to 180 min. Following the heat treatment, cells were placed at 4°C overnight prior to assaying viability. Dead cells were obtained by submerging the cells in liquid nitrogen followed by rapid thawing for several cycles or by boiling the cells for 5 min at 100°C.

### Determination of viability

Following each stress treatment, 0.25 g fresh weight of cells for each designated condition (triplicates) were incubated in 12.5 mL of culture medium at 25°C in the dark. After 7 days incubation, the final fresh weight of cells in the exponential growth phase was determined. Under these growth conditions, rice cells showed a linear relationship (r = 0.989) between the final fresh weight and the cell inoculum fresh weight ranging from 0.03 to 0.34 g [7,18].

TTC reduction assays were conducted as described previously [18]. Briefly, following freeze-thaw cycles, cells (0.3 g) were washed with 5 mL of sterile water for 3–4 h (this washing process was necessary to avoid erroneously high survival values) before being placed in 4 mL of TTC solution (0.08% TTC in 0.05 M potassium phosphate buffer, pH 7.5) for 24 h at 25°C in the dark. Following removal of TTC solution, the cells were extracted with 5 mL of 95% ethanol for 2 days and absorbance read at 485 nm.

Freezing tolerance was represented as the LT_50_, the lethal temperature at which there was a 50% decrease in the survival compared to the non-stressed control (TTC, regrowth tests), unless otherwise specified. All viability assays were done in triplicate.

### Determination of cell growth

Following incubation of rice suspension cultures under the designated conditions, cells were harvested by filtration on a mesh filter, rinsed well with 250 mL of water and blotted on paper towel for 1 min before determination of fresh weight. Dry weights were determined after oven-drying at 70°C for 2 days.

### Determination of water content and osmolarity

Following incubation of rice suspension cultures at 25°C for 7 days with or without 75 μM ABA, cells were harvested by filtration on a mesh filter, rinsed well with 250 mL of water. The cells were placed into a syringe and centrifuged at 1500 rpm for 10 min to remove extracellular water before determination of cell fresh weight and osmolarity. Dry weight of a part of the centrifuged cells was determined by oven-drying at 70°C for 2 days and water content was expressed on a dry weight basis. The syringe with the remainder of the cells was placed into LN and rewarmed at room temperature before being squeezed to obtain the cell sap. The cell sap was centrifuged at 10000 rpm for 10 min and osmolarity of the supernatant was determined with a vapor pressure osmometer (Wescor, Inc).

### Microscopic observation of rice suspension cells

Rice suspension cells existed as cell clumps (each clump composed of ca. 50–100 cells). This made it difficult to observe the morphological structure of individual cells except for the cells in the peripheral zone of a clump, which might represent only a portion of structural changes. Instead, we observed protoplasts isolated from the cell clumps for morphological studies. The aseptically harvested rice cells following incubation with or without 75 μM ABA at 25°C for 7 days were used for protoplast isolation. Cells were incubated in medium containing 0.5 M sorbitol, 5 mM MES (pH 5.5), 1 mM CaCl_2_, 1% (w/v) Cellulase R-10 (Kinki Yakuruto) and 0.1% (w/v) Pectolyase (Seishin Pharmaceutical) in a Petri dish at room temperature for 10 h. Following gentle agitation, the medium was passed through a nylon mesh to obtain protoplast solution. Following washing with 0.5 M sorbitol, 1 mM CaCl_2_, the protoplasts were observed under microscopy and photographed. The cell morphology of 150 or more protoplasts for each treatment was classified into three categories based on the extent of vacuolation and augmentation of cytoplasm (Figure 9).

### Freeze tests for rice seedlings

Rice (*O. sativa* L. cv. Nipponbare) seeds were hulled and sterilized with 10% (v/v) sodium hypochlorite solution (active chlorine concentration: ca 1%) for 15 min and grown aseptically on semi-solid MS medium containing 0.8% (w/v) agar and 3% (w/v) sucrose for 8 days under light conditions (50 μmol s^-1^ m^-2^) at 25°C. Then they were grown in the presence of 75 μM ABA for additional 4 days or 1–7 days at 25°C before used for determination of freezing tolerance. Seedlings grown for 9 days from seeds without ABA were used as control plants.

Rice seedlings (five for each test temperature) grown as described above were rinsed well with sterilized water, then trimmed to have about 5 cm of shoots and 1 cm of roots and wrapped with water-soaked Kimwipe sheet. They were placed in 50 mL pre-sterilized plastic centrifuge tubes with 1 mL of water in the bottom. All these processes were done aseptically in a laminar flow chamber. The tubes were placed in a cool-bath held at −1°C, ice-inoculated with the liquid nitrogen cooled rods. After double-checking the freezing of the water inside the tube and held there for 1 h before being cooled by 2°C/h to - 5°C. At designated temperatures (−3 and −5°C), the tubes were taken out from the bath and placed at 4°C for thawing. Then the tubes with plants were incubated at 25°C for 14 days before determining visually the regrowth and injuries.

To determine freezing tolerance of leaves, they were excised from seedlings grown *in vitro* as described above with or without ABA. The excised leaves were wrapped with water-soaked Kimwipe sheet and placed in 15 mL pre-sterilized tubes. Following incubation at −1°C, they were ice-inoculated and held there for 30 min, then cooled by 2°C/h to designated temperatures. Following slow thawing at 4°C, 3 mL of sterile water was added to each test tube and incubated overnight at 25°C before determining the conductivity of the leachate (C_1_) using a Horiba conductivity meter DS-12. Conductivity of the same samples after autoclaving at 105°C for 1 min was also measured (C_2_). Percentage leakage was calculated from (C_1_-C_w_) / (C_2_-C_w_), where C_w_ was the conductivity of water.

### Experimental design and data presentation

The experiments were replicated three times or more and the data are presented as the mean ± SD. When necessary, Tukey-Kramer multiple comparison tests were performed to show statistically significant differences at 5% level (Figures 1, 5, 8 and 9).

### Definitions

Cold hardiness, resistance to subfreezing temperatures by tolerating extracellular freezing (freezing tolerance) or by avoiding freezing (such as transient and deep supercooling); cold acclimation, acquisition of cold hardiness in response to non-lethal low temperatures alone or in combination with short photoperiods; chilling temperatures, low temperature ranges above 0°C (e.g., 0-10°C); chilling injury, injury caused by exposure to chilling temperatures; chilling-sensitive or tolerant, sensitive or tolerant to chilling temperatures.

## Abbreviations

ABA: Abscisic acid; LT50: 50% killing temperature; TTC: 2,3,5-triphenyl tetrazolium chloride.

## Competing interests

The authors declare that they have no competing interests.

## Authors’ contributions

RS, AM, KA and RM contributed equally to this work by conducting most of the freeze tests and by optimizing the ABA-induced freezing tolerance in rice cultures and seedlings. HM studied the cold hardiness of rice seedlings. KK participated in coordination and supervision. MI conceived of the study, supervised the study and drafted the manuscript. All authors read and approved the final manuscript.
